# Trend of the Tuberculous Pleurisy Notification Rate in Eastern China During 2017-2021: Spatiotemporal Analysis

**DOI:** 10.2196/49859

**Published:** 2023-10-30

**Authors:** Ying Zhou, Dan Luo, Kui Liu, Bin Chen, Songhua Chen, Junhang Pan, Zhengwei Liu, Jianmin Jiang

**Affiliations:** 1 School of Public Health Hangzhou Normal University Hangzhou China; 2 School of Public Health Hangzhou Medical College Hangzhou China; 3 Department of Tuberculosis Control and Prevention Zhejiang Provincial Center for Disease Control and Prevention Hangzhou China; 4 National Centre for Tuberculosis Control and Prevention Chinese Center for Disease Control and Prevention Beijing China; 5 Key Laboratory of Vaccine Prevention and Control of Infectious Disease of Zhejiang Province Zhejiang Provincial Center for Disease Control and Prevention Hangzhou China

**Keywords:** tuberculous pleurisy, spatio-temporal, epidemiology, prediction, time series

## Abstract

**Background:**

Tuberculous pleurisy (TP) presents a serious allergic reaction in the pleura caused by *Mycobacterium tuberculosis*; however, few studies have described its spatial epidemiological characteristics in eastern China.

**Objective:**

This study aimed to determine the epidemiological distribution of TP and predict its further development in Zhejiang Province.

**Methods:**

Data on all notified cases of TP in Zhejiang Province, China, from 2017 to 2021 were collected from the existing tuberculosis information management system. Analyses, including spatial autocorrelation and spatial-temporal scan analysis, were performed to identify hot spots and clusters, respectively. The prediction of TP prevalence was performed using the seasonal autoregressive integrated moving average (SARIMA), Holt-Winters exponential smoothing, and Prophet models using R (The R Foundation) and Python (Python Software Foundation).

**Results:**

The average notification rate of TP in Zhejiang Province was 7.06 cases per 100,000 population, peaking in the summer. The male-to-female ratio was 2.18:1. In terms of geographical distribution, clusters of cases were observed in the western part of Zhejiang Province, including parts of Hangzhou, Quzhou, Jinhua, Lishui, Wenzhou, and Taizhou city. Spatial-temporal analysis identified 1 most likely cluster and 4 secondary clusters. The Holt-Winters model outperformed the SARIMA and Prophet models in predicting the trend in TP prevalence.

**Conclusions:**

The western region of Zhejiang Province had the highest risk of TP. Comprehensive interventions, such as chest x-ray screening and symptom screening, should be reinforced to improve early identification. Additionally, a more systematic assessment of the prevalence trend of TP should include more predictors.

## Introduction

Humanity has been affected by tuberculosis (TB) for centuries, which is an infectious agent–caused disease considered the leading cause of mortality worldwide since the beginning of recorded history. Among people who contract TB each year, the majority are adults, with the incidence of TB being higher in men than in women. According to the Global Tuberculosis Report of 2022 released by the World Health Organization, the estimated TB incidence rate in 2021 increased by 3.6% from 2020, and China accounted for 7.4% of TB cases worldwide. Furthermore, the number of TB deaths worldwide successively increased from 1.4 million in 2019 to 1.6 million in 2021, only after the onset of the COVID-19 pandemic [1]. *Mycobacterium tuberculosis* generally affects not only the lungs but also other sites such as the pleura [2].

Tuberculosis pleurisy (TP) is a common clinical manifestation of TB post primary infection or due to the reactivation of latent TB [3]. Available documentation shows that TP accounts for about 4% of total tuberculosis cases in the United States and Brazil, 20% of cases in South Africa, and 7.3% of cases in Korea [4]. In the United States, the incidence of both TP and TB has been declining, but to a lesser degree for TP [5]. In 2018, it was categorized as pulmonary tuberculosis (PTB) in China [6]. TP can be divided into dry and exudative pleurisy. Dry pleurisy is an early inflammatory reaction of the pleura, usually without obvious imaging abnormalities. Exudative pleurisy is characterized by unilateral or bilateral pleural effusions, which often occur as thickened pleural adhesions with slow absorption [7]. The recommended standard regimen for TP is up to 8 months, which is longer than the usual treatment period for TB [8]. Previous studies have shown that the prevalence of TP is low in women, while the number of patients with TP is higher in eastern China, among farmers and patients with AIDS [9-11]. However, the temporal periodicity and spatial clustering characteristics of TP have not been explored in eastern China.

Zhejiang Province is located in the eastern region of China and has a developed economy. In recent years, certain parts of the province have experienced a high burden of TB. Understanding the spatial and temporal patterns of TB incidence can provide insights into the clusters of this disease [12]. Spatial-temporal analysis has been widely used to describe the epidemiological distribution and aggregation of infectious diseases in both space and time; however, few studies have explored the spatial epidemiological characteristics of TP. In comparison to traditional epidemiological techniques, tools using geographic information systems have significant advantages in tracking disease, identifying areas of clustering, and analyzing the spread of disease across communities [13]. Spatiotemporal cluster detection has played a crucial role in understanding infectious disease spread, with its dynamic course and dimensions of time and place, and a combination of both [14]. In addition, time-series models were selected to provide scientific clues for predicting the prevalence of diseases such as desiccation syndrome and AIDS [15,16]. However, there are few such studies on TP.

Therefore, this study aimed to use spatial-temporal analysis to identify the epidemiological characteristics of TP. Furthermore, the spatial differentiation of TP can provide a visual map depicting the diversity of TP occurrence across different regions. Additionally, this study used time-series models to provide scientific clues for the control of and interventions targeting TP infections in the province.

## Methods

### Overview of the Study Area

Zhejiang Province is located on the eastern coast of China, with a land area of 101,800 km^2^, accounting for 1.06% of the country’s land area [17]. The province encompasses plains, mountains, coasts, islands, and lakes. Zhejiang Province comprises 90 counties and cities, including 2 subprovincial cities and 9 prefecture-level cities [17,18].

### Data Source

All recorded cases of TP in Zhejiang Province from 2017 to 2021 were obtained from the TB Information Management System (TBIMS), which is a first-generation web-based information system designed and built by the National Tuberculosis Control and Prevention Center in 2005 [19]. The system collected data on demographics, diagnosis, laboratory results, and treatment outcomes of all patients with TB during the specified period. In addition, demographic data for Zhejiang Province were obtained from the China Information System for Disease Control and Prevention.

### Definition

Diagnostic criteria for TP were as follows: (1) confirmed patients: cultures of pleural biopsy specimens were positive, and the strain was identified as *M tuberculosis*, changes in tuberculosis confirmed through pleural tissue biopsy; and (2) clinically diagnosed patients: imaging examination showing pleural effusion with (i) a moderately positive or strongly positive outcome on the tuberculin pure protein derivative skin test, (ii) a positive outcome on the γ-interferon release test, or (iii) a positive outcome on the *M tuberculosis* antibody test [20]. Delays in seeking medical treatment were indicated when the interval between the appearance of the first symptom and the visit to the designated hospital was > 2 weeks, and the delay in diagnosis was the time between the first consultation and a confirmed diagnosis [21].

### General Epidemiological Characteristics of Patients With TP

The features of patients with TP in Zhejiang Province from 2017 to 2021 are described in the context of sex, age group, city, antituberculosis treatment, mode of case finding, treatment history, delay in access to care and final diagnosis, and treatment outcomes.

### Spatial Autocorrelation Analysis

In this study, the Moran I index and local indicators of spatial association (LISA) were used to analyze global and local spatial autocorrelation. The global Moran I index is a comprehensive measure of spatial autocorrelation in the whole study area, and it can indicate the average degree of spatial differences between each unit and the surrounding area. If partial positive and partial negative spatial correlations coexist in the overall study area, LISA are needed to reveal the possible spatial variability [22].

### Space-Time Scan Statistics

For the spatiotemporal analysis, we used the Kulldorff spatial-temporal scan statistic to identify clusters based on specific time and location combinations. This statistic uses a Poisson probability distribution mode. The Kulldorff method uses a moving cylindrical window to scan the study area. The height of the cylinder represents the possible clustering time, the bottom represents the clustering area, and the radius serves as the scanning risk population [23]. The significance of the clusters identified at a 95% confidence level was tested using Monte Carlo simulations to identify the clusters.

### Prediction Models

Three models were used in this study. The Holt-Winters exponential smoothing model eliminated some random fluctuations, which can assign different weights to the data of each period and reasonably predict the future development trend [16]. The seasonal autoregressive integrated moving average (SARIMA) model divided the observed value into 3 parts: residuals, seasonal feature, and true trend [24]. The Holt-Winters exponential smoothing model and SARIMA model were introduced more specifically in a previous study [16]. The Prophet model, developed by Meta, allowed using time as a regressor to fit both linear and nonlinear functions of time [25], and a more specific introduction to this model is provided in Xie et al’s [26] study.

### Statistical Analysis

Descriptive analysis was conducted using Excel (Microsoft Corp) and SPSS (version 28; IBM Corp). The Moran I index was calculated using GeoDa (version 1.20), and SaTScan software (version 10.1; Harvard Medical School) was used to determine the spatial and temporal aggregation of TP. All visual results are presented using the ArcGIS software (version 10.8; SERI Inc). The prediction of TP prevalence was model-fitted using R (version 4.2.0; The R Foundation) and Python (version 3.8; Python Software Foundation).

### Ethical Considerations

This study was approved by the Ethics Committee of the Zhejiang Provincial Center for Disease Control and Prevention (audit number 2022-032-01), which waived the requirement for informed consent. All personal information obtained in this study was treated and kept confidential in accordance with the required protocols.

## Results

### General Epidemiological Characteristics of Patients With TP

**Figure 1:**
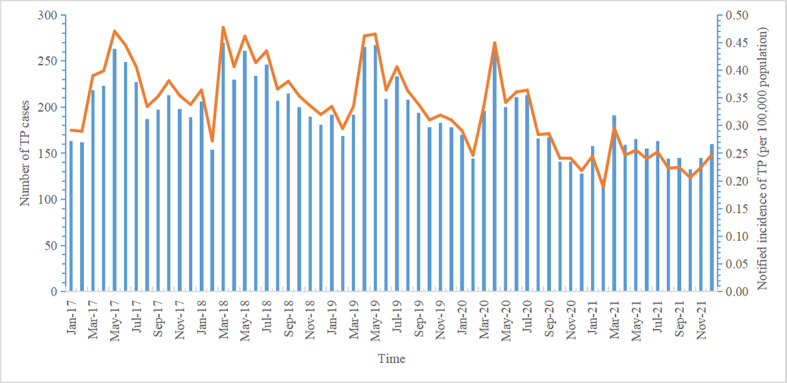
Change in the trend of tuberculous pleurisy notification rate and case numbers by month.

During the study period, a total of 11,531 patients with TP were identified, with a notification rate of 7.8 cases per 100,000 population in 2017, 8.2 cases per 100,000 population in 2018, 7.7 cases per 100,000 population in 2019, 6.6 cases per 100,000 population in 2020, and 5.0 cases per 100,000 population in 2021, respectively (Figure 1).The male to female ratio was 2.18:1. Among all cases of TP, the top notified cases and notification rate were noted in 2018 (n=2594; 8.2 cases per 100,000 population). The number of cases peaked in the second quarter (n=3354) and plummeted in the fourth quarter (n=2558) during the study period, indicating seasonal distribution characteristics. In terms of age distribution, 74 (0.64%) individuals were aged 0-18 years, 6359 (55.15%) were aged 19-59 years, and 5098 (44.21%) were aged ≥60 years. In terms of geographical distribution, 2319 (20%) cases were notified in Hangzhou, while 1294 (11.22%), 1393 (12.08%), 1452 (12.59%), and 1259 (10.92%) cases were reported in Wenzhou, Ningbo, Jinhua, and Taizhou, respectively. Passive findings was the main source of TP identification in Zhejiang Province. Nearly all patients (n=11,326, 98.22%) were treated for the first time, with nearly 90% of them having completed treatment. Delayed access to medical care was observed among 6317 (54.78%) patients with TP, and diagnosis was delayed among 1462 (12.68%) patients. In addition, 86.26% (n=9949) of patients with TP were treated with a duration of ≥9 months (Table 1).

**Table 1:** Epidemiological characteristics of patients with tuberculosis pleurisy in Zhejiang Province from 2017 to 2021 (N=11,531).

Characteristic		Patients, n (%)
**Sex**		
	Male	7901 (68.52)
	Female	3630 (31.48)
**Age group (years)**		
	0-18	74 (0.64)
	19-59	6359 (55.15)
	≥60	5098 (44.21)
**City**		
	Hangzhou	2319 (20.11)
	Jinhua	1452 (12.59)
	Ningbo	1393 (12.08)
	Wenzhou	1294 (11.22)
	Taizhou	1259 (10.92)
	Shaoxing	1078 (9.35)
	Quzhou	858 (7.44)
	Jiaxing	674 (5.85)
	Huzhou	582 (5.05)
	Lishui	506 (4.39)
	Zhoushan	116 (1.00)
**Case finding**		
	Passive finding	11,513 (99.84)
	Active finding	18 (0.16)
**Treatment history**		
	Initial treatment	11,326 (98.22)
	Retreatment	205 (1.78)
**Anti** **–** **tuberculosis pleurisy treatment**		
	Yes	11,523 (99.93)
	No	8 (0.07)
**Time of delay in seeking medical treatment (days)**		
	0-14	5214 (45.22)
	15-29	2968 (25.74)
	30-44	1569 (13.61)
	45-59	503 (4.36)
	≥60	1261 (10.94)
	Unknown	16 (0.14)
**Time of delay in diagnosis in the health system** **(days)**		
	0-14	10,069 (87.32)
	15-29	773 (6.70)
	30-44	302 (2.62)
	45-59	122 (1.06)
	≥60	237 (2.06)
	Unknown	28 (0.24)
**Interval between diagnosis of tuberculosis pleurisy and end of treatment (days)**		
	0-180	734 (6.37)
	181-270	795 (6.89)
	≥271	9949 (86.28)
	Unknown	53 (0.46)
**Treatment outcome**		
	Treatment completed	10,422 (90.38)
	Death	203 (1.76)
	Cure	242 (2.10)
	Changes in diagnosis	204 (1.77)
	Failure	14 (0.13)
	Adverse reactions	122 (1.06)
	Transfer to multidrug-resistant tuberculosis pleurisy treatment	10 (0.09)
	Others	314 (2.73)

### Spatial Autocorrelation Analysis

**Figure 2:**
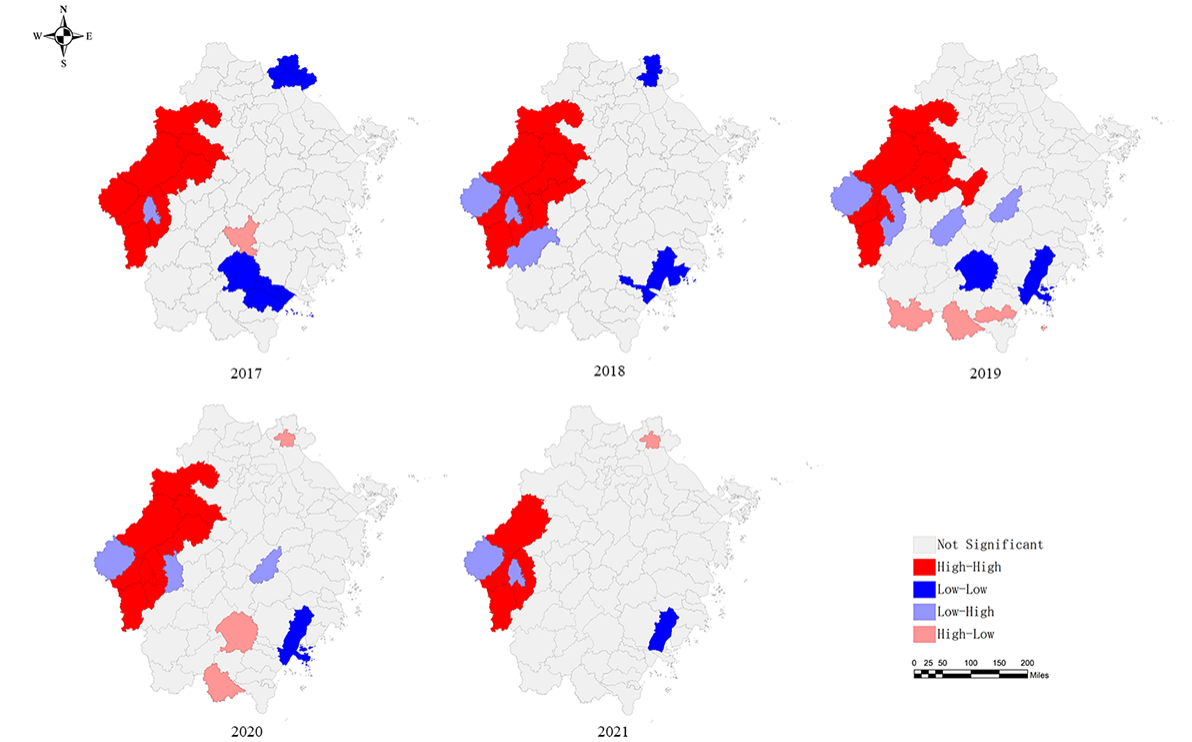
Local indicators of spatial association of the tuberculosis pleurisy notification rate in Zhejiang Province.

On spatial autocorrelation analysis, the global Moran I index ranged from 0.187 to 0.362, indicating spatial heterogeneity in the distribution of patients with TP in Zhejiang Province from 2017 to 2021. Furthermore, there was a positive correlation between the notification rate and spatial distribution (*P*<.01; Table 2). The local autocorrelation LISA cluster plot showed that in Zhejiang Province, hot spot areas were located in parts of Quzhou, Jinhua, and Hangzhou, and cold spot areas were clustered in Jiaxing, Wenzhou, and Lishui. Although the hot and cold spots changed every year, Jiangshan, Qujiang, Changshan in Quzhou City, and Chun'an in Hangzhou City emerged as hot spots throughout the study period. Furthermore, Longwan and Yueqing in Wenzhou City were identified as cold spots in 4 out of the 5 years (Figure 2).

**Table 2:** Spatial autocorrelation analysis of the tuberculosis pleurisy notification rate.

Year	Moran I index	*z* score	*P* value
2017	0.362	5.275	.001
2018	0.296	4.099	.001
2019	0.187	2.632	.007
2020	0.209	3.026	.006
2021	0.241	3.864	.003

### Spatial-Temporal Scan Statistics

**Figure 3:**
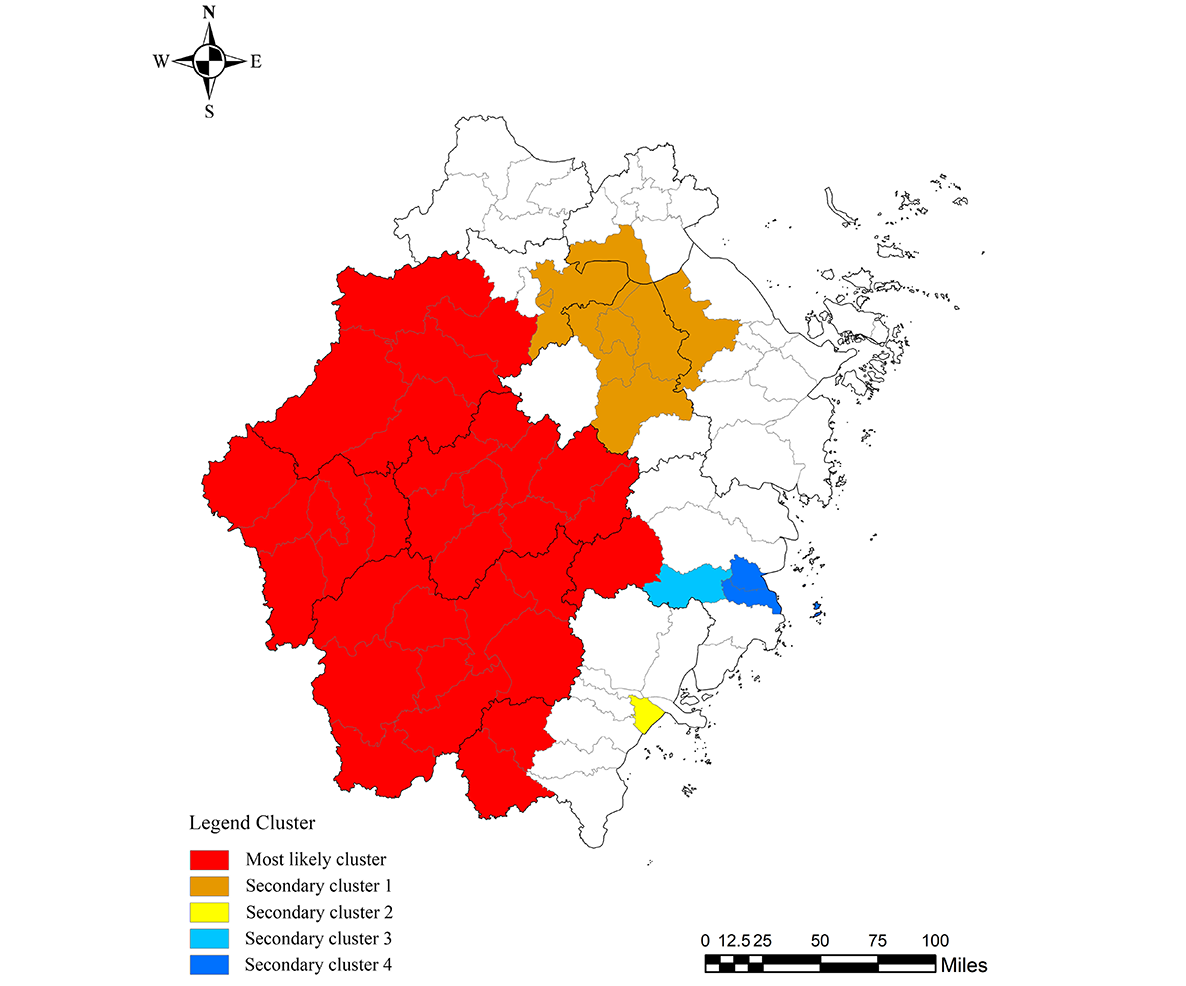
Spatial-temporal clustering of the tuberculosis pleurisy notification rate in Zhejiang Province.

Spatial-temporal scan statistics of TP in Zhejiang Province from 2010 to 2021 identified 1 most likely cluster and 4 secondary clusters. The most likely cluster was the high-risk cluster, which encompassed 32 counties in western and southwestern Zhejiang Province, including all counties and districts of Quzhou, Jinhua, and Lishui; Wencheng and Taishun in Wenzhou; and Xianju in Lishui. The most likely cluster period ranged from April 2017 to September 2019, with 2383 cases of TP and a relative risk of 1.98 (log likelihood ratio=382.55, *P*<.01; Table 3 and Figure 3).

**Table 3:** Spatial-temporal scanning of the tuberculosis pleurisy notification rate in Zhejiang Province, 2017-2021.

Cluster type	Cluster period	Coordinates/radius	Counties, n	Notified (expected) cases, n (n)	Log likelihood ratio	Relative risk	*P* value
Most likely cluster	January 20, 2017, to September 30, 2019	(28.578400° N, 118.595300° E)/199.19 km	32	2383 (1341.84)	382.55	1.98	<.01
Secondary cluster 1	January 1, 2017, to June 30, 2018	(30.042100° N, 120.663400° E)/51.20 km	8	785 (488.22)	80.06	1.65	<.01
Secondary cluster 2	February 1, 2018, to July 31, 2020	(27.885900° N, 120.815300° E)/0 km	1	48 (3.87)	76.84	12.46	<.01
Secondary cluster 3	February 1, 2017, to November 30, 2018	(28.602000° N, 121.076900° E)/0 km	1	124 (48.34)	41.40	2.58	<.01
Secondary cluster 4	April 1, 2017, to August 31, 2018	(28.652100° N, 121.475800° E)/12.06 km	2	129 (74.78)	16.25	1.73	<.01

### Model Predictions

On comparing the fitness of predicting TP prevalence trends in 2021, the Holt-Winters exponential smoothing model showed better predictive performance than the SARIMA and Prophet models, with lower mean-square error, root-mean-square error, mean absolute error, mean absolute percentage error, and symmetric mean absolute percentage error values (Table 4). The composition of the original sequence and the predictive results determined using the Holt-Winters exponential smoothing model are presented in Table 4. The exponential smoothing model was used to predict the number of notified cases of TP, and its actual values in 2021 are shown in Table 5.

**Table 4:** Critical indices of the Holt-Winters exponential smoothing, SARIMA^a^, and Prophet models.

Indices	SARIMA	Holt-Winters	Prophet
MSE^b^	978.593	479.170	579.114
RMSE^c^	31.282	21.890	24.065
MAE^d^	25.247	17.380	17.501
MAPE^e^	16.270	11.130	N/A^f^
SMAPE^g^	16.883	12.080	N/A

^a^SARIMA: seasonal autoregressive integrated moving average.

^b^MSE: mean-square error.

^c^RMSE: root-mean-square error.

^d^MAE: mean absolute error.

^e^MAPE: mean absolute percentage error.

^f^N/A: not applicable.

^g^SMAPE: symmetric mean absolute percentage error.

**Table 5:** Holt-Winters exponential smoothing model used to predict the number of notified cases of tuberculosis pleurisy and its actual values in 2021.

Month	Actual value	Estimate	95% CI
January	158	141	104-178
February	122	119	105-133
March	191	162	161-163
April	159	176	134-217
May	165	175	140-210
June	155	156	116-197
July	163	156	132-181
August	144	129	99-159
September	145	128	116-140
October	133	120	82-157
November	145	115	108-121
December	160	108	107-108

## Discussion

### Principal Findings

TP, as a subgroup of PTB in China, has attracted increasing attention in recent years due to its severity. According to a bibliometric study, there has been an increasing number of TP-related studies in the past decade, and China had the most studies in this field [27]; furthermore, Mexico, the Philippines, Vietnam, China, and India had the highest prevalences of TP worldwide [5]. Zhejiang Province is a developed coastal area of China, and the notification rate of TP accounts for a certain proportion. Therefore, it is necessary to understand cluster epidemics at the spatial and temporal levels.

In this study, epidemiological characteristics showed that men were more likely to develop TP than women, which is in line with the results of previous studies [28,29]. Given that TP occurs secondary to PTB, we speculated that possible explanations for sex differences were attributed to lifestyle and genetic susceptibility, such as smoking, alcohol use, drug abuse, sex hormones, and genetic variations [30,31]. Therefore, it is necessary to consider men as the key population for prevention and control, such as by strengthening screening and improving the awareness of TP in this specific population. Furthermore, we found that the TP notification rate peaked mainly in the summer. As shown in the literature, when the mean ambient temperature increased from 15.1 °C to 24.5 °C, the cumulative adjusted relative risk of hospitalization for TB was 1.07 (95% CI 1.00-1.14) [32]. Although there is no clear evidence showing a direct correlation between TP and temperature, we postulate that due to the increased temperature, there is a likely increase in the synthesis of mycolic acids, which are vital for the growth of *M tuberculosis* and contribute to its subverting of and escaping from the immune system [33].

In our study, 6317 (54.78%) patients with TP experienced delayed access to medical care. A literature analysis of TB cases from 32 provinces in China showed that patient delays were also common among TB cases, with a median of 20 (IQR 6-46) days [21]. We speculated that a comparatively long patient delay may result in further invasion of the pleura. Furthermore, the median delay in PTB diagnosis in China was 1 (IQR 0-8) day, while a delay of TP diagnosis of more than 14 days accounted for 12.68% of cases in our study [21]. To our knowledge, a definite diagnosis of TP was made by detecting *M tuberculosis* from pleural effusion or pleural tissue [34]. However, culturing *M tuberculosis* takes 2-8 weeks to obtain results in some regions without access to rapid diagnosis tools, which could influence effective medical interventions [35]. Consequently, further delay in anti-TB treatment for TP might result in pleural thickening or TB empyema that requires surgical resolution [36]. Considering the long duration of the disease, insidious onset, and lack of specificity of diagnostic methods for TP [37,38], it is prone to both abovementioned delays, which may further lead to increased transmission of TB [39]. Therefore, timely detection, such as using x-ray imaging or computed tomography, and adequate health education are needed to identify TP in the early stages and improve awareness of TB information [40]. For patients with normal immune function, the current recommended treatment duration is 6 months for isoniazid and rifampicin with 2 months for ethambutol and pyrazinamide [8]. In China, the National Tuberculosis Program guidelines specifically recommend that the treatment period for TP be ≥9 months, while nearly 14% of patients had received insufficient treatment. Therefore, more attention must be paid to the treatment duration of TP.

Spatial-temporal analysis is commonly used to explore disease clusters and identify areas at high risk of infectious disease [41]. Spatial analysis indicated spatial heterogeneity among patients with TP, with hot spots located mainly in the western cities of Zhejiang Province. The relatively large agricultural population and relatively low economic status in these areas, along with limited knowledge related to TP, are the common risk factors for the development of TP [42]. Therefore, the early detection strategy of TP in this population should be strengthened by implementing modern approaches, such as large-scale active screening. Furthermore, the spatial-temporal analysis indicated that the most likely clusters were mainly concentrated in the western part of Zhejiang Province, which were similar to the results of spatial analysis. This result unveils the distribution pattern of TP incidence in Zhejiang Province and provides a theoretical basis for the regional rational allocation of health resources related to TP and effective implementation of comprehensive prevention and control measures [41]. Therefore, exploring the causes and mechanisms leading to the regional clustering of TP should be carried out in the future.

Each predictive model has its own limitations and merits due to the different prerequisites and mechanisms involved in predicting epidemics. In this study, our exponential smoothing model has a good prediction effect, and the actual value of 66.7% falls within the 95% CI of the predicted value. Part of the actual value that is not within the prediction range may be unsatisfactory for clinical applications. The possible reason was the impact of the COVID-19 pandemic, resulting in the deviation of the model prediction.

### Limitations

However, this study also has some limitations. First, TP is not easy to diagnose, and it was not mandatory to include TBIMS before 2018, which could cause an underestimation of TP prevalence from our available surveillance system. Second, the combination and breakdown of different areas of the panel data makes it impossible to carry out detailed analysis in some areas. Third, the prediction model did not include other meteorological factors such as humidity and wind. Last, some potential influencing factors were not considered, such as HIV coinfection, which would be considered in the future.

### Conclusions

In this study, the male population had a high notification rate of TP, and a high notification rate of this disease was noted during summer. The western region of Zhejiang Province has the highest relative risk of TP. Comprehensive interventions, such as chest x-ray screening and symptom screening, should be strengthened to improve early identification. Additionally, a more systematic assessment of the prevalence trend of TP should include more predictors.

## Abbreviations

LISA: local indicators of spatial association
PTB: pulmonary tuberculosis
SARIMA: seasonal autoregressive integrated moving average
TB: tuberculosis
TP: tuberculous pleurisy

## Data Availability

All data and materials were included in this paper. The corresponding author (JJ) can provide data upon reasonable request after all studies and substudies have been completed.

## References

[1] (2022). Global Tuberculosis Report 2022. World Health Organization.

[2] Boonsarngsuk V, Mangkang K, Santanirand P (2018). Prevalence and risk factors of drug-resistant extrapulmonary tuberculosis. Clin Respir J.

[3] Ryan H, Yoo J, Darsini P (2017). Corticosteroids for tuberculous pleurisy. Cochrane Database Syst Rev.

[4] Jeon D (2014). Tuberculous pleurisy: an update. Tuberc Respir Dis (Seoul).

[5] Baumann MH, Nolan R, Petrini M, Lee YCG, Light RW, Schneider E (2007). Pleural tuberculosis in the United States: incidence and drug resistance. Chest.

[6] People’s Republic of China state health and Family Planning Commission (2018). Tuberculosis classification (WS196—2017). Electronic J Emerg Infect Dis.

[7] National Health and Family Planning Commission of the People's Republic of China (2018). Diagnostic criteria for tuberculosis. Electronic J Emerg Infect Dis.

[8] Shaw JA, Koegelenberg CFN (2021). Pleural Tuberculosis. Clin Chest Med.

[9] Frye MD, Pozsik CJ, Sahn SA (1997). Tuberculous pleurisy is more common in AIDS than in non-AIDS patients with tuberculosis. Chest.

[10] Wen P, Wei M, Han C, He Y, Wang M (2019). Risk factors for tuberculous empyema in pleural tuberculosis patients. Sci Rep.

[11] Chen S, Wang Y, Zhan Y, Liu C, Wang Q, Feng J, Li Y, Chen H, Zeng Z (2023). The incidence of tuberculous pleurisy in mainland China from 2005 to 2018. Front Public Health.

[12] Mohamed AS, Levine M, Camp JW, Lund E, Yoder JS, Glickman LT, Moore GE (2014). Temporal patterns of human and canine Giardia infection in the United States: 2003-2009. Prev Vet Med.

[13] Gao S, Mioc D, Anton F, Yi X, Coleman DJ (2008). Online GIS services for mapping and sharing disease information. Int J Health Geogr.

[14] Rogers DJ, Randolph SE (2003). Studying the global distribution of infectious diseases using GIS and RS. Nat Rev Microbiol.

[15] Luo Z, Jia X, Bao J, Song Z, Zhu H, Liu M, Yang Y, Shi X (2022). A combined model of SARIMA and prophet models in forecasting AIDS incidence in Henan Province, China. Int J Environ Res Public Health.

[16] Wang S, Wei F, Li H, Wang Z, Wei P (2022). Comparison of SARIMA model and Holt-Winters model in predicting the incidence of Sjögren's syndrome. Int J Rheum Dis.

[17] Liu K, Li T, Vongpradith A, Wang F, Peng Y, Wang W, Chai C, Chen S, Zhang Y, Zhou L, Chen X, Bian Q, Chen B, Wang X, Jiang J (2020). Identification and prediction of tuberculosis in Eastern China: analyses from 10-year population-based notification data in Zhejiang Province, China. Sci Rep.

[18] Liu K, Chen S, Zhang Y, Li T, Xie B, Wang W, Wang F, Peng Y, Ai L, Chen B, Wang X, Jiang J (2022). Tuberculosis burden caused by migrant population in Eastern China: evidence from notification records in Zhejiang Province during 2013-2017. BMC Infect Dis.

[19] Li T, Du X, Shewade HD, Soe KT, Zhang H (2018). What happens to migrant tuberculosis patients who are transferred out using a web-based system in China?. PLoS One.

[20] Huang Yi, Zuo Lei, Ultrasound Professional Committee of Tuberculosis Branch of Chinese Medical Association, Interventional Ultrasound Professional Committee of Interventional Physician Branch of Chinese Medical Doctor Association (2022). Expert consensus on ultrasound diagnosis, classification and interventional therapy of tuberculous pleurisy (2022 Edition). Chinese Journal of Antituberculosis.

[21] Li T, Du X, Kang J, Luo D, Liu X, Zhao Y (2023). Patient, diagnosis, and treatment delays among tuberculosis patients before and during COVID-19 epidemic - China, 2018-2022. China CDC Wkly.

[22] Li Meifang, OU Jinpei (2016). Spatio-temporal analysis of influenza A(H1N1) in China during 2009-2013 based on GIS. Geogr Res.

[23] Yamamura M, de Freitas IM, Santo Neto M, Chiaravalloti Neto F, Popolin MAP, Arroyo LH, Rodrigues LBB, Crispim JA, Arcêncio Ricardo Alexandre (2016). Spatial analysis of avoidable hospitalizations due to tuberculosis in Ribeirao Preto, SP, Brazil (2006-2012). Rev Saude Publica.

[24] Ndeh NT, Tesfaldet YT, Budnard J, Chuaicharoen P (2022). The secondary outcome of public health measures amidst the COVID-19 pandemic in the spread of other respiratory infectious diseases in Thailand. Travel Med Infect Dis.

[25] Ogallo W, Wanyana I, Tadesse GA, Wanjiru C, Akinwande V, Kabwama S, Remy SL, Wachira C, Okwako S, Kizito S, Wanyenze R, Kiwanuka S, Walcott-Bryant A (2023). Quantifying the impact of COVID-19 on essential health services: a comparison of interrupted time series analysis using Prophet and Poisson regression models. J Am Med Inform Assoc.

[26] Xie C, Wen H, Yang W, Cai J, Zhang P, Wu R, Li M, Huang S (2021). Trend analysis and forecast of daily reported incidence of hand, foot and mouth disease in Hubei, China by Prophet model. Sci Rep.

[27] Bian Y, Deng M, Zhang Q, Hou G (2022). Global trends of research on tuberculous pleurisy over the past 15 years: a bibliometric analysis. Front Cell Infect Microbiol.

[28] Tahseen S, Khanzada FM, Baloch AQ, Abbas Q, Bhutto MM, Alizai AW, Zaman S, Qasim Z, Durrani MN, Farough MK, Ambreen A, Safdar N, Mustafa T (2020). Extrapulmonary tuberculosis in Pakistan- A nation-wide multicenter retrospective study. PLoS One.

[29] Luo Y, Yan F, Xue Y, Mao L, Lin Q, Tang G, Song H, Wu S, Ouyang R, Yuan X, Liu W, Yu J, Zhou Y, Hou H, Sun X, Wang F, Sun Z (2020). Diagnostic utility of pleural fluid T-SPOT and interferon-gamma for tuberculous pleurisy: a two-center prospective cohort study in China. Int J Infect Dis.

[30] Khan AH, Sulaiman SAS, Hassali MA, Khan KU, Ming LC, Mateen O, Ullah MO (2020). Effect of smoking on treatment outcome among tuberculosis patients in Malaysia; a multicenter study. BMC Public Health.

[31] Kirenga BJ, Ssengooba W, Muwonge C, Nakiyingi L, Kyaligonza S, Kasozi S, Mugabe F, Boeree M, Joloba M, Okwera A (2015). Tuberculosis risk factors among tuberculosis patients in Kampala, Uganda: implications for tuberculosis control. BMC Public Health.

[32] Chong KC, Yeoh EK, Leung CC, Lau SYF, Lam HCY, Goggins WB, Zhao S, Ran J, Mohammad KN, Chan RWY, Lai CKC, Chan PKS, Leung CSY, Chen VXY, Wang Y, Wei Y (2022). Independent effect of weather, air pollutants, and seasonal influenza on risk of tuberculosis hospitalization: an analysis of 22-year hospital admission data. Sci Total Environ.

[33] Takayama K, Armstrong EL, Davidson LA, Kunugi KA, Kilburn JO (1978). Effect of low temperature on growth, viability, and synthesis of mycolic acids of Mycobacterium tuberculosis strain H37Ra. Am Rev Respir Dis.

[34] Jeon D (2014). Tuberculous pleurisy: an update. Tuberc Respir Dis (Seoul).

[35] Ritchie SR, Harrison AC, Vaughan RH, Calder L, Morris AJ (2007). New recommendations for duration of respiratory isolation based on time to detect Mycobacterium tuberculosis in liquid culture. Eur Respir J.

[36] Barbas CS, Cukier A, de Varvalho CR, Barbas Filho JV, Light RW (1991). The relationship between pleural fluid findings and the development of pleural thickening in patients with pleural tuberculosis. Chest.

[37] Liu YJ, Jiang Z, Chen H, Jing H, Cao X, Coia J E, Song Z (2020). Description of demographic and clinical characteristics of extrapulmonary tuberculosis in Shandong, China. Hippokratia.

[38] Lee C, Chiu L, Chang C, Chung F, Li S, Chou C, Wang C, Lin S (2022). The clinical experience of Mycobacterial culture yield of pleural tissue by pleuroscopic pleural biopsy among tuberculous pleurisy patients. Medicina (Kaunas).

[39] Zhongwei J, Zuhong L (2016). Effect of delayed diagnosis and treatment on transmission of tuberculosis. J Syst Sci Math Sci.

[40] Jiang J, Zhang X, Wang F (2020). Influencing factors for the delay in seeking health care of pulmonary tuberculosis in the elderly. Prev Med.

[41] Qian Y, Kun C (2013). Spatial distribution patterns of pulmonary tuberculosis incidence in Zhejiang province: spatial autocorrelation analysis. Chin J Public Health.

[42] Gui JJ, Zhang TF, Liu ZF (2016). Epidemiological characteristics and spatial clusters of pulmonary tuberculosis in Zhejiang province,2005-2011. Chin J Public Health.

